# Alginate-Based Carriers Loaded with Mulberry (*Morus alba* L.) Leaf Extract: A Promising Strategy for Prolonging 1-Deoxynojirimicyn (DNJ) Systemic Activity for the Nutraceutical Management of Hyperglycemic Conditions

**DOI:** 10.3390/molecules29040797

**Published:** 2024-02-08

**Authors:** Lucia Marchetti, Eleonora Truzzi, Maria Cecilia Rossi, Stefania Benvenuti, Silvia Cappellozza, Alessio Saviane, Luca Bogataj, Cristina Siligardi, Davide Bertelli

**Affiliations:** 1 Department of Life Sciences, University of Modena and Reggio Emilia, Via G. Campi 103, 41125 Modena, Italy; lucia.marchetti@unimore.it (L.M.); stefania.benvenuti@unimore.it (S.B.); 2 Department of Chemical and Geological Sciences, University of Modena and Reggio Emilia, Via G. Campi 103, 41125 Modena, Italy; 3 Centro Interdipartimentale Grandi Strumenti, University of Modena and Reggio Emilia, Via G. Campi 213/A, 41125 Modena, Italy; mariacecilia.rossi@unimore.it; 4 Council for Agricultural Research and Economics, Research Centre for Agriculture and Environment (CREA-AA), Via Eulero, 6a, 35143 Padova, Italy; silvia.cappellozza@crea.gov.it (S.C.); alessio.saviane@crea.gov.it (A.S.); luca.bogataj@crea.gov.it (L.B.); 5 Department of Engineering “Enzo Ferrari”, University of Modena and Reggio Emilia, 41125 Modena, Italy; cristina.siligardi@unimore.it

**Keywords:** alginate, 1-deoxynojirimicyn (DNJ), NMR relaxation time constants, HPLC-MS, ATR-FTIR spectroscopy, swelling studies

## Abstract

The iminosugar 1-deoxynojirimicyn (DNJ) contained in mulberry leaves has displayed systemic beneficial effects against disorders of carbohydrate metabolism. Nevertheless, its effect is impaired by the short half-life. Alginate-based carriers were developed to encapsulate a DNJ-rich mulberry extract: Ca-alginate beads, obtained by external gelation, and spray-dried alginate microparticles (SDMs). Mean size and distribution, morphology, drug loading, encapsulation efficiency, experimental yield, and release characteristics were determined for the two formulations. Ca-alginate beads and SDMs exhibited an encapsulation efficiency of about 54% and 98%, respectively, and a DNJ loading in the range of 0.43–0.63 μg/mg. The in vitro release study demonstrated the carriers’ capability in controlling the DNJ release in acid and basic conditions (<50% in 5 h), due to electrostatic interactions, which were demonstrated by 1H-NMR relaxometry studies. Thus, alginate-based particles proved to be promising strategies for producing food supplements containing mulberry leaf extracts for the management of hyperglycemic state.

## 1. Introduction

Diabetes mellitus (DM) is a complex metabolic disorder that affects the natural regulation of blood glucose levels. This condition is characterized by chronic hyperglycemia and disturbances of carbohydrate and fat metabolism, resulting from inadequate insulin secretion (type 1 DM) or insulin resistance (type 2 DM). Type 2 DM is becoming a major health concern and the incidence is on the rise worldwide according to the World Health Organization [1]. Several studies have shown that the use of phytochemicals from natural sources is a suitable and promising approach for the management of diabetes, especially at the early stage of the disease [2]. Mulberry (*Morus* spp. L.) leaf extract has been traditionally used as a functional food in Asian countries to treat DM [3,4,5] and several food supplements containing *M. alba* leaf extract are currently available in the European market. *M. alba* extract can be considered one of the most promising functional foods for the treatment of hyperglycemic conditions and prevention of long-term complications in diabetic patients due to the synergistic effect of the various phytochemicals. The beneficial effects of *M. alba* leaf extract are related to the presence of polyphenols and the iminosugar 1-deoxynojirimicyn (DNJ). The polyphenols kaempferol, quercetin, and chlorogenic acid have been demonstrated to exert beneficial effects against the progression of DM by decreasing the generation of advanced glycation products (AGEs) [6]. Additionally, DNJ is a well-documented inhibitor of the intestinal α-glycosidases capable of slowing down the rate of polysaccharides’ conversion into sugars [7,8]. Additionally, DNJ was demonstrated to exert positive systemic effects against the hyperglycemic state by down-regulating the expression of glucose transporters and increasing the mRNA expression of hepatic enzymes involved in glucose metabolism [5]. Finally, DNJ improves insulin sensitivity and activates fatty acids’ β-oxidation in vivo [3]. Unfortunately, DNJ’s systemic effects are impaired by its short half-life due to the high hydrophilicity. As previously reported, DNJ is quickly eliminated from the body by renal excretion [9]. Thus, frequent administrations of DNJ are required to reach the therapeutic dose.

The employment of delivery systems might represent a valid strategy for enhancing the biological effect of therapeutic agents. In particular, the encapsulation of bioactive compounds allows the control of their release profile in the biological environment by means of the chemical and physical features of the delivery carriers.

The present work aimed at the entrapment of mulberry crude extract in alginate-based (ALG) particles for prolonging the DNJ release. The anionic polymer ALG was chosen for its high biocompatibility, biodegradability, and its common employment as a food additive and entrapping material in the pharmaceutical field [10,11,12,13]. The polymer ALG is a generic name assigned to a series of unbranched polyanionic polysaccharides, composed of β-D-mannuronic acid (M) and α-L-guluronic acid (G) units, linked by a 1 → 4 glycosidic bonds [12]. One of the most convenient features of ALG is the capacity to cross-link in water solutions by chelation of divalent cations (Ba^2+^, Ca^2+^, Mg^2+^) from pendant carboxylic moieties. The cross-linking is generated when a divalent cation is coordinated by four guluronate units, thus introducing intermittent binding between the linear ALG chains. This peculiar behavior is also known as ionic gelation, and it is one of the easiest ways to encapsulate bioactive agents [11,12]. Two different strategies were attempted to encapsulate the DNJ-rich mulberry extract (EXT). The first consisted of the development of Ca-ALG millimetric-size particles (Ca-ALG beads), while the second in the obtainment of sodium ALG spray-dried microparticles (SDMs).

Hence, the cross-linking of ALG was selected as the first strategy to form a reticulated structure capable of retaining the hydrophilic compound. Conversely, spray-drying was chosen as the second strategy to directly encapsulate DNJ into polymeric microspheres, being a rapid, scalable, and one-step process. Both approaches were designed to be potentially applied in peroral delivery. To our best knowledge, there are no studies devoted to the formulation of Ca-ALG beads or ALG SDMs containing mulberry EXT as a novel therapeutic approach for hyperglycemia. The main goal was to design and produce an ideal delivery system to maintain a constant intestinal concentration of DNJ for prolonging its in vivo bioactivity. The proposed delivery strategies might be employed to produce more efficacious food supplements for the treatment of the hyperglycemic condition. The two presented techniques are simple and cost-effective. The experimental conditions were set up and optimized to achieve a standardized product.

## 2. Results and Discussion

In this work, two novel approaches were designed and developed for encapsulating the mulberry main active constituent using ALG as polymer. The main goal was to develop a delivery system able to efficiently entrap the DNJ and retard its release in the gut, where it could directly act as a glucosidase inhibitor and reach the circulation to exert systemic effects. The encapsulation of small hydrophilic molecules is a big challenge since poor entrapment efficiency and undesired release profiles are often obtained. ALG was selected as polymer to overcome these issues and improve the retention of DNJ due to its negatively charged groups that might interact with the positively charged iminogroup of DNJ. Millimetric Ca-ALG beads were selected for their low surface-area-to-volume ratio, which can reduce the release of small hydrophilic drugs [14]. Conversely, micrometric SDMs were considered for their easy production and scalable technology at the industrial level. Spray-drying technology is rapid, continuous, highly reproducible, and allows the obtainment of a product with low moisture content. An ALG polymer with a high G/M ratio was selected. Indeed, the specific G conformation guarantees a high degree of coordination of bivalent ions, leading to the formation of a more rigid gel. Therefore, the particles composed of guluronic-rich alginates might result in more effective systems for a controlled diffusion release [15,16].

### 2.1. Determination of DNJ by HPLC-ESI-TQ-MS Analysis

**Figure 1:**
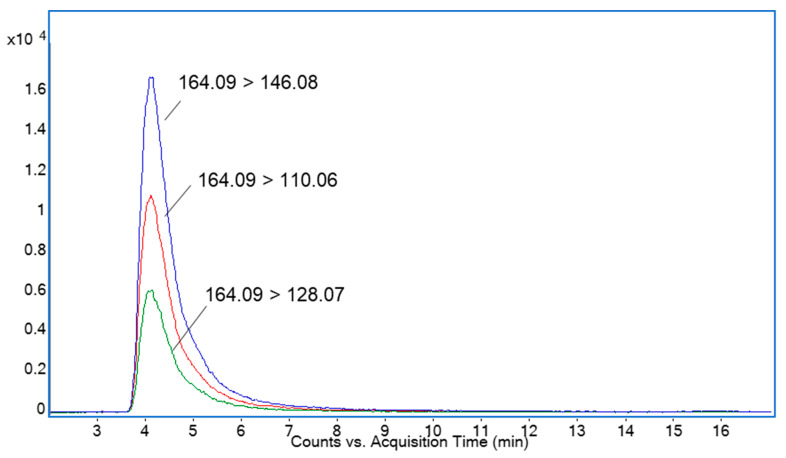
An overlay of MRM transitions monitored for DNJ.

HILIC coupled with mass spectrometry (MS) has proven to be one of the most recommended methods for DNJ determination due to the shortest duration of this analysis and its highest sensitivity [17,18,19]. The quantitative approach was based on multiple reaction monitoring (MRM). The transition of the intense molecular ion (*m*/*z* 164.09) to the product ions (*m*/*z* 146.08, 128.07, 110.06) for subsequent losses of water molecules (18 Da) was used for selective DNJ determination. Figure 1 shows the MRM chromatogram of mulberry leaf ethanolic EXT. The amount of DNJ in the ethanolic EXT was 15.93 ± 0.19 μg/mL and 1.93 ± 0.04 mg/g in the freeze-dried product. The dry product yield of the freeze-drying process was 4.50 ± 0.52 mg/mL.

### 2.2. Particle Size, Morphology, and DNJ Content

The particle size and drug content parameters of Ca-ALG beads and SDMs are reported in Table 1. DNJ content is expressed as entrapment efficiency (EE%) and drug loading (DL).

**Figure 2:**
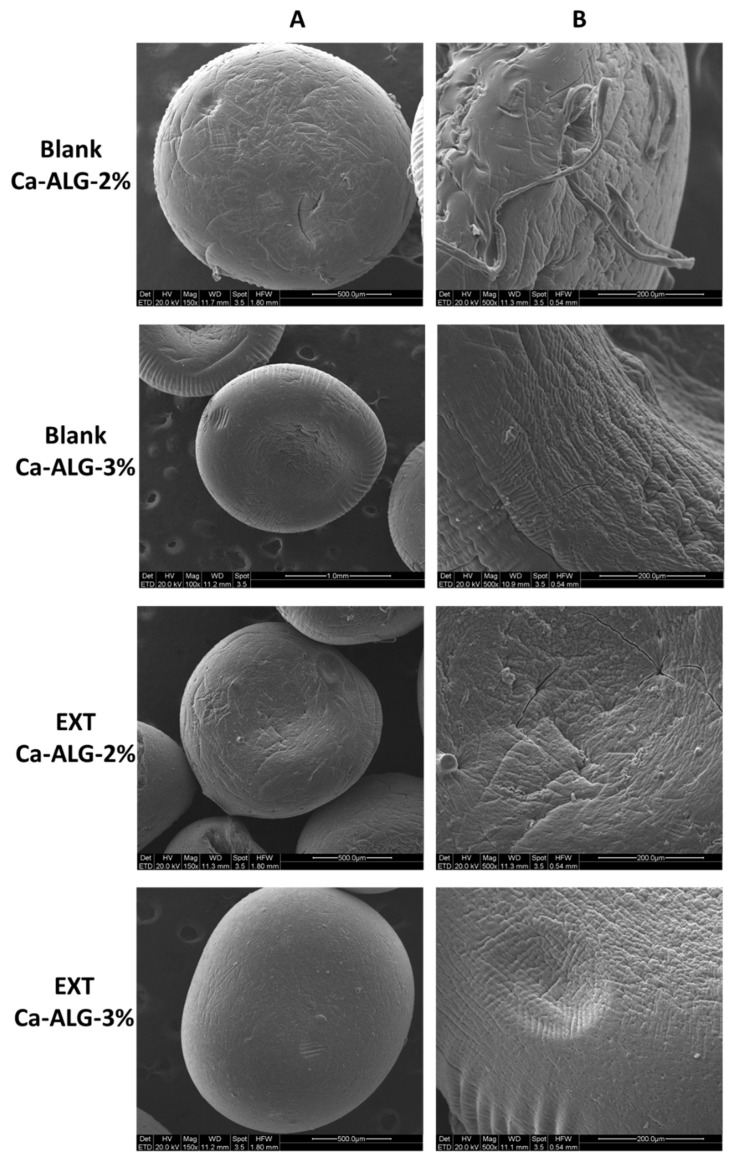
ESEM micrograph of blank and EXT-loaded ALG beads 2 and 3% at 100 or 150× (**A**) and 500× (**B**) magnification.

Ca-ALG beads had spherical geometry and good size homogeneity which implies a monodisperse population of particles. Both 2 and 3% blank Ca-ALG beads showed a slightly higher mean diameter than their respective unloaded formulation. Nonetheless, bead size homogeneity was not significantly affected by the addition of the extract, maintaining the narrow distribution for both concentrations. No significant difference in size was observed either between 2 and 3% Ca-ALG beads, suggesting that the small variation in ALG concentration did not impact the particle dimensions. Dry EXT was also solubilized in CaCl_2_ solution in the same proportion as for ALG solution to attempt the maximum efficiency in DNJ encapsulation [20]; however, a quite low EE% of DNJ was achieved in the beads due to the extremely high water solubility, hydrophilic nature, and low molecular weight. A critical loss of compound could have been caused by the final washing step to remove the excess cross-linking agent [11,21]. Furthermore, DNJ might have been lost during the drying process, when the water incorporated in the gelled beads was removed and partially absorbed on filter paper. In the case of Ca-ALG-3% beads, a higher EE% was found (*p* < 0.05) due to the higher concentration of Na-ALG employed in the formulation process. The increase in ALG concentration provided a greater availability of binding sites for Ca^2+^ ions, leading to the formation of a more compact gel membrane with a smaller pore size [13]. Thus, the leak of DNJ during the formulation process was moderate [22,23]. The morphological analysis confirmed the roundness of the beads and highlighted some differences in the surface structure mainly depending on the polymer concentration. Indeed, the particle surface of blank-Ca-ALG-2% appeared rough and less compact than blank-Ca-ALG-3%. The encapsulation of the EXT seemed to confer a more compact surface texture (Figure 2).

**Figure 3:**
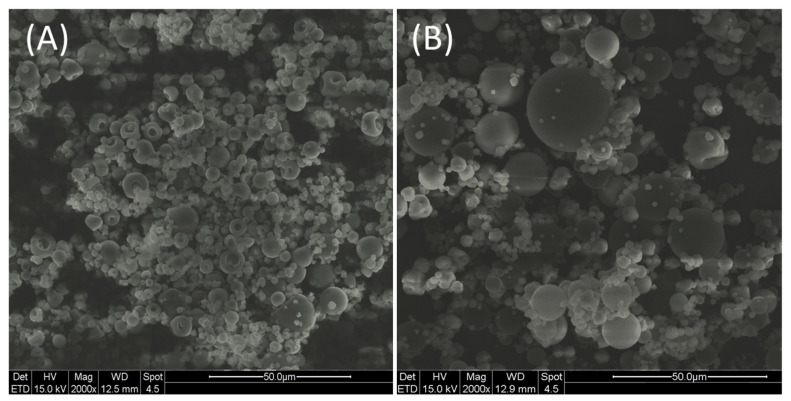
ESEM micrographs of blank SDMs (**A**) and SDMs (**B**) at 2000× magnification.

Regarding the SDMs, the yield was quite low. Conventional spray dryers at the laboratory scale have been reported to be not very satisfactory due to the loss of particles onto the walls of the drying chamber and cyclone [24]. The blank SDMs exhibited a monodisperse population with a low span value. Conversely, SDMs showed a bimodal distribution (Table 1). This evidence may be attributable to an increased propensity of particles to form agglomerates in the presence of crude EXT, due to the natural occurrence of sugars, glycosides, and other sticky components [25,26]. As was previously pointed out, microparticles encapsulating plant extracts usually tend to establish bridges to connect to each other by absorbing moisture from the environment [26]. The non-homogeneity of SDMs was also highlighted by the ESEM analysis (Figure 3). Some morphological differences were observed between blank (Figure 3A) and EXT-loaded SDMs (Figure 3B).

Loaded microparticles (EXT-SDMs) displayed a spherical shape and smooth surface. Conversely, some of the blank SDMs have collapsed or appeared as dimpled spheres. According to the hypothesis proposed by Ameri et al., the formation of a polymer film at the external surface of the droplet during the initial drying phase caused by the rapid evaporation of solvent may explain the presence of dimples. The further increase in the concentration of the polymer at the surface could hamper the water diffusion to the periphery of the droplet and cause a build-up of vapor pressure inside the particle. Finally, the bursting of the film would result in dimples or drilled particles [27]. Regarding the encapsulation of DNJ in SDMs, the efficiency was almost complete, suggesting that the ratio between polymer and EXT and the spray-drying parameters were optimal [28]. The DL of SDMs was significantly higher (*p* < 0.01) than beads’ DL. Notwithstanding SDMs exhibiting a two-fold higher EE%, the DL was of the same order of magnitude as those of beads. The preparation of SDMs involved the use of the liquid EXT without any preliminary concentration. Therefore, the ratio between DNJ and ALG in SDMs was lower than in Ca-ALG beads.

### 2.3. Fourier-Transform Infrared (FTIR) Spectroscopy

**Figure 4:**
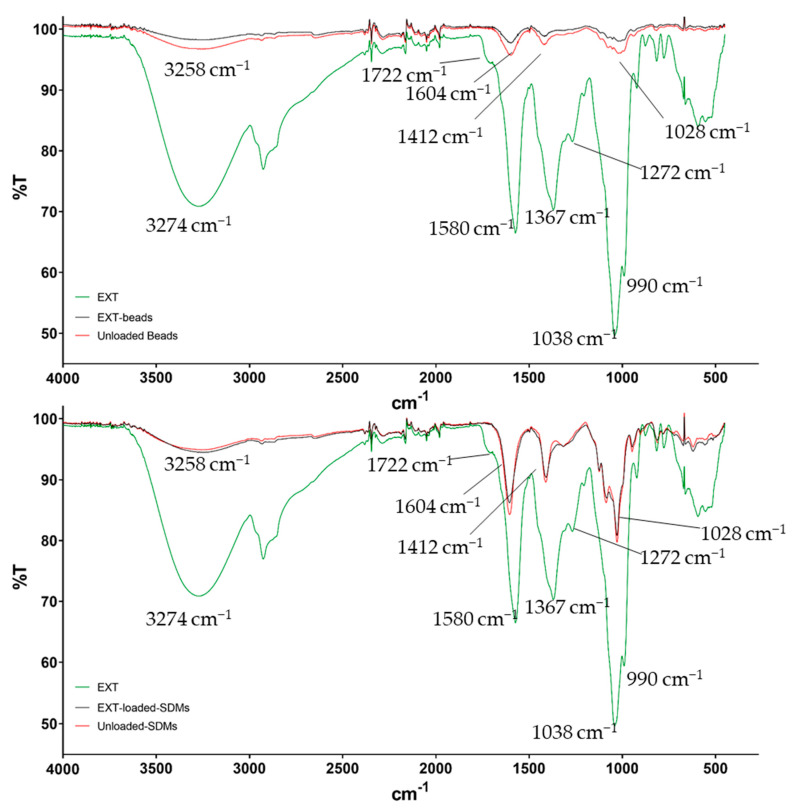
FTIR spectra of mulberry extract (EXT), blank, and EXT-loaded Ca-ALG-2% beads (**top**) and SDMs (**bottom**).

The attenuated total reflectance Fourier-transform infrared (ATR-FTIR) spectra of mulberry leaf EXT, Ca-ALG beads, SDMs, and corresponding blanks were recorded and are displayed in Figure 4. Since no differences between Ca-ALG-2% and -3% were recorded, only the spectra of Ca-ALG-2% beads are reported. The spectrum of the raw EXT showed several broad peaks, mainly ascribable to functional groups of phenolic acids and flavonoids. Specifically, the spectral bands at 3274, 1038, and 990 cm^−1^ were representative of O–H and C–O stretching of alcohols, while the peaks at 1367 and 1272 cm^−1^ were induced by phenol O–H bends and secondary O–H in-plane bends, respectively. Finally, the signals at 1722 and 1580 cm^−1^ were attributable to C=O bonds of carboxylic and carbonylic groups of ketones or esters [29,30]. In regard to the unloaded SDMs and the unloaded Ca-ALG beads, the main signals around 3258, 1604, 1412, and 1028 cm^−1^ were induced by stretching vibrations of O–H, C=O (symmetrical and asymmetrical) of carboxylate groups, and C–O–C bonds, respectively [10]. These spectra were similar with slight shifts of about 20 cm^−1^ at lower wavelengths in the case of Ca-ALG beads for the hydroxyl and carbonylic vibrations. The shifts were caused by the interaction of these functional groups with calcium ions [10,31]. The loading of the EXT into both Ca-ALG beads and SDMs did not change the IR spectra of the polymer, and the typical signal bands of the EXT were not detected. This evidence was previously reported by Bagheri et al. and corroborates that the encapsulation of the EXT successfully took place [10].

### 2.4. Interaction Study by NMR Relaxometry

**Figure 5:**
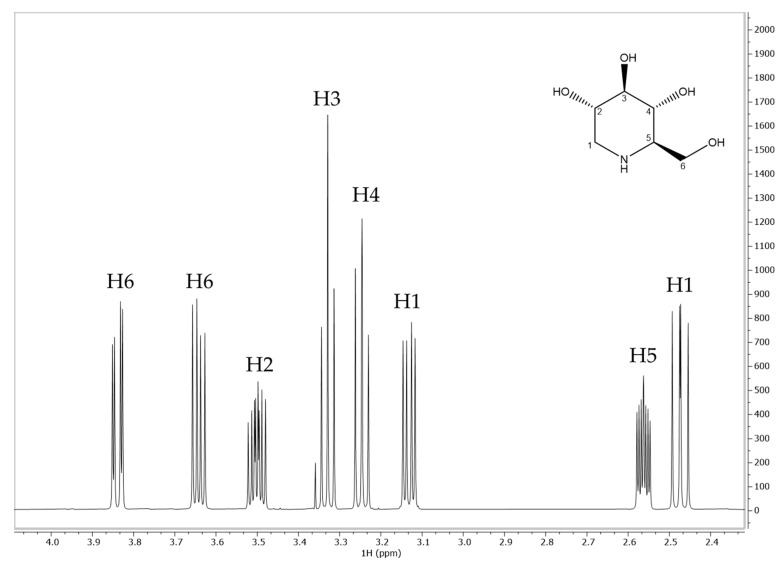
^1^H-NMR spectrum and assignments of DNJ in deuterium oxide (D_2_O). Chemical shifts are related to TSP.

A proton nuclear magnetic resonance (^1^H-NMR) relaxometry study was carried out to investigate the nature of the interaction between DNJ and ALG in depth. In particular, the monitoring and measurement of spin–lattice relaxation rates can provide information about ligand mobility in the presence or absence of the macromolecule [32,33,34]. ^1^H-NMR chemical shifts, signal multiplicity, and assignments of DNJ are reported below in Figure 5 and Table 2.

As can be seen from Table 3, the presence of ALG caused a remarkable decrease in the T_1_ρ values of DNJ protons, especially for H_2_, H_3_, H_4,_ and H_5_. This decrease indicated the reduction in the mobility of the small molecule [35,36], suggesting the formation of electrostatic interactions, hydrogen bonding, or other weak intermolecular forces between DNJ protons and carboxyl groups of ALG. Once it was established that the interaction occurred, NMR was further employed for the determination of binding constants, according to the method proposed by [32]. When a ligand (L) interacts with a macromolecule (M) an equilibrium occurs, and it is expressed by the following equation:

L+M⇄LM

If the complex is held together by weak intermolecular forces, the equilibrium constant is defined as an association constant (K_a_) and the product has chemical characteristics which still strongly resemble the unassociated (or free) molecules. K_a_ can be obtained as:

$$K_{a}=\frac{\left[LM\right]}{\left[L\right]\left[M\right]}$$

where [L], [M], and [LM] are the molar concentrations of the free ligand, macromolecule, and complex, respectively.

Relaxation rate (R) is the reciprocal of T_1_, and it is the NMR experimental parameter that best explains the dynamic coupling between L and M. The experimentally determined relaxation rate of the ligand in the presence of the macromolecule (R_obs_) can be expressed as:

$$R_{obs}=p_{f}R_{f}+p_{b}R_{b}$$

p_f_: fraction of the free ligand;
p_b_: fraction of the associated ligand;
R_f_: relaxation rate in the free state;
R_b_: relaxation rate in the associated state.

Assuming that the fraction of the free ligand is far larger than that of the associated form

(*p_b_* <<<1), and taking p_b_ + p_f_ = 1:

(1) $$∆R=R_{obs}-R_{f}≅p_{b}R_{b}$$

The association constant K_a_ can be expressed as:

(2) $$K_{a}=\frac{\left[LM\right]}{\left[L\right]\left[M\right]}=\frac{\left[LM\right]}{\left[L\right]\left(\left[M_{0}\right]-\left[LM\right]\right)}$$

(3) $$\left[LM\right]=\frac{K_{a}\left[L\right]M_{0}}{1+K_{a}\left[L\right]}$$

where [M_0_] is the initial concentration of the macromolecule.

Since p_b_ is the fraction of the associated ligand, then:

(4) $$p_{b}=\frac{\left[LM\right]}{\left[L\right]+\left[LM\right]}≅\frac{\left[LM\right]}{\left[L\right]}$$

Finally, by substitution of (3) in (4) and then in (1), the following equation is obtained:

(5) $$\frac{1}{∆R}=\left(\frac{1}{K_{a}}+\left[L\right]\right)\frac{1}{R_{b}\left[M_{0}\right]}$$

Equation (5) shows a linear relationship for 1/∆R and [L]. In practice, T_1_ values were measured at different DNJ concentrations (4–25 mM) in the absence and presence of ALG (1% w/v). Then, the difference between the reciprocals of T_1_ was calculated (∆R). The value (1/∆R) was plotted versus DNJ concentrations, and a linear regression was obtained for each proton. For 1/∆R = 0, −1/K_a_ was extrapolated (Table 4 and Table 5).

It is generally accepted that NMR-based determinations of K_a_ are only reliable if in the range 10–10^4^ M^−1^ [35]. The obtained values of K_a_ were all within the acceptability range and consistent with those reported by Di Cocco et al. between ALG and different amino acids, which are similar to DNJ from a chemical point of view [32]. The highest interaction constants were obtained for H_6_ (109 M^−1^), H_3_ (120 M^−1^), H_2_ (141 M^−1^), and H_4_ (151 M^−1^). This evidence suggested that a specific interaction occurred between DNJ and ALG protons, due to the most significant decrease in the observed relaxation times. Data related to T_1_ measurements (Table 5) also agreed with the T_1_ρ values reported above in Table 3. DNJ is a basic iminosugar (pKa 8.06) that might be electrostatically bonded to the carboxylic groups of guluronic and mannuronic acids. This electrostatic interaction may contribute to the stabilization of the EXT-loaded particle and achieve the prolonged release of the active compound, regulated by a dynamic equilibrium of dissociation from the polymer.

### 2.5. Ca-ALG Bead Swelling Study

**Figure 6:**
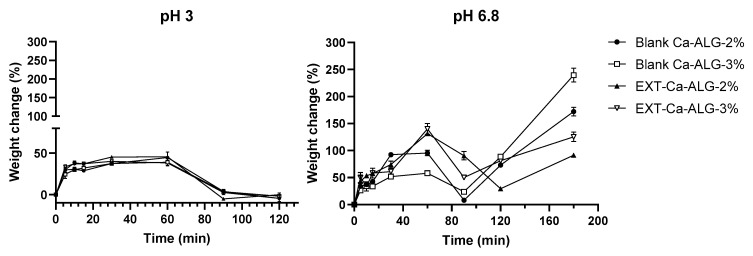
Dynamic uptake of water from blank and EXT-loaded Ca-ALG beads in simulated gastric (pH 3) and intestinal (pH 6.8) fluid. Data are shown as means ± SD of three independent experiments.

The swelling study of Ca-ALG beads was performed in simulated gastric and intestinal fluids to understand the mechanism of bead degradation after oral administration. The pH of the aqueous media was selected to mimic the post-prandial conditions of the gastrointestinal tract. The swelling ratio of the beads was heavily dependent on the pH and ionic strength of the solution in which the particles were placed (Figure 6). The weight of all the bead samples moderately increased in simulated gastric fluid (around 40–45%), reaching the maximum swelling in approximately 60 min. This evidence suggested that the beads were stable in the acid environment. The increment of the weight was induced by the hydration of the hydrophilic groups of ALG [37] due to the water penetration through the bead surface and the filling of the pores among the polymer chains. No significant differences were noticed between the preparations.

Concerning the simulated intestinal environment, all the formulations exhibited more significant swelling rates. The beads showed a fast water uptake in the first 60 min followed by a weight loss due to the progressive external erosion induced by the dissolution of alginate chains after ion exchange (up to 90 min). The first water uptake was mainly related to the exchange between Na^+^ and Ca^2+^ ions linked to the external monomer units [38]. Indeed, the polymer chains undergo relaxation after ion exchange, leading to significant water absorption and bead swelling.

Afterward, a massive water uptake (from 90–120 min until the end) occurred prevailing over the erosion mechanism. The increase in the weight was induced by the exchange of Na^+^ from the medium with the Ca^2+^ linked to the carboxylic groups of the polyguluronate units of the particle core, as previously described by Bajpai and co-workers [38]. One-way ANOVA and Tukey’s post hoc test highlighted significant differences among all the samples at 180 min in the simulated intestinal fluid (*p* < 0.01). Specifically, the highest swelling degree was obtained for Ca-ALG-3% beads, thus indicating that the concentration of the polymer was positively correlated with an increased relaxation of the ALG chains. On the other hand, in EXT-Ca-ALG beads the overall swelling was significantly reduced (*p* < 0.0001), supporting the hypothesis that the electrostatic interaction between ALG and DNJ may stabilize the structure by hardening polymer chains.

### 2.6. In Vitro Release of DNJ

**Figure 7:**
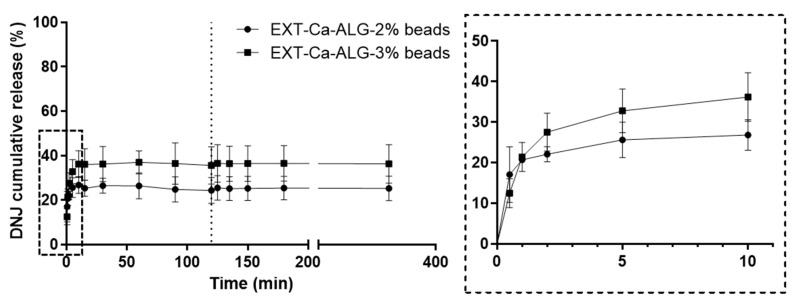
In vitro DNJ release from Ca-ALG beads in simulated gastric (0–120 min) and intestinal (120–360 min) fluids (separated by the dotted line). Data in graphs are shown as mean ± SD of three independent experiments. The enlargement of the dotted-line area shows the release profile in the first minutes.

**Figure 8:**
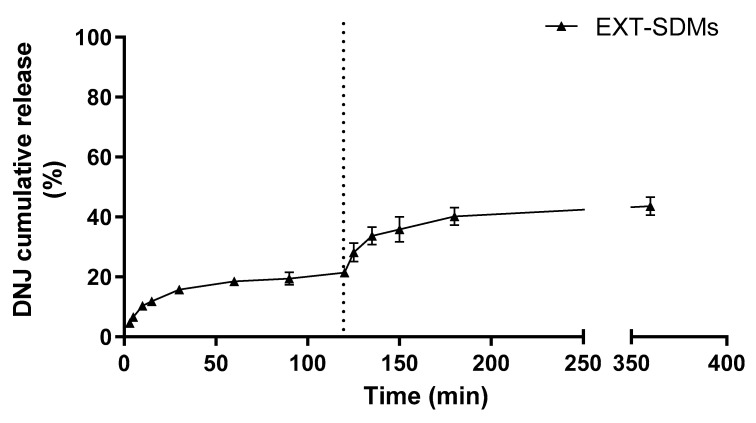
In vitro DNJ release from SDMs in simulated gastric (0–120 min) and intestinal (120–360 min) fluids (separated by the dotted line). Data in graphs are shown as mean ± SD of three independent experiments.

The release of DNJ from Ca-ALG beads and SDMs was investigated in the simulated gastrointestinal fluids (Figure 7 and Figure 8) under the same conditions as the swelling study. In the case of Ca-ALG beads, a burst effect was observed in the first 10 min upon contact with gastric fluid, reaching 28 ± 4 and 36 ± 6% of the cumulative release for 2% and 3% ALG concentrations, respectively. The initial release of DNJ from Ca-ALG-3% beads was slightly faster than that from Ca-ALG-2% beads, even though higher ALG concentrations were generally reported to be more effective in retarding the drug release [39,40,41]. This initial rapid release was probably caused by the fast permeation of water among the polymer chains observed during the swelling studies. Thus, the DNJ absorbed into the outer polymer chains via electrostatic interaction quickly dissolved in the external medium [42]. Subsequently, the DNJ release in acid conditions was slow and constant up to the first 2 h, which is the approximate residence time in the stomach. DNJ was likely released by diffusion from the insoluble matrix since the swelling of Ca-ALG beads in acid conditions only occurred to a minor extent [43]. Conversely, the DNJ release in the intestinal fluid was expected to be induced by the complex process of “swelling–dissolution–erosion” [43], according to the significant loss of weight recorded during the swelling studies. Interestingly, the DNJ concentration did not increase over 27 ± 6 and 37 ± 6% for Ca-ALG-2% and Ca-ALG-3% beads, respectively, even though the progressive disintegration and solubilization of the beads occurred. Considering the extremely high solubility and small chemical structure of DNJ, the low amount released in the environment could be explained by the strong interaction with the polymer demonstrated by the NMR studies.

The in vitro release was also performed for SDMs under the same conditions. In this case, the DNJ release was carried out by using a dialysis bag to avoid the withdrawal of the microparticles from the aqueous media. The release profile of DNJ from SDMs was comparable to that from the beads (Figure 8), suggesting that it is not governed by the structure of the carrier. Ordinarily, the release of drugs from Na-ALG is related to the solubility of the drug and the polymer, the polymer concentration, and the interaction among them [44]. Here, the main factor involved in controlling the DNJ release from SDMs seemed to be the interaction between the polymer and the iminosugar. Furthermore, the pH of the solvents might have played a central role in controlling the release of DNJ. Na-ALG might undergo structural changes depending on the pH. In acid conditions (pH < 5), the carboxylic groups are protonated, converting the sodium alginate to alginic acid and leading to the shrinking of the polymer. Thus, the SDMs were not altered by the media during the release at pH 3 [45]. The cumulative percentage gradually increased from 6 ± 2 to 20 ± 2% in simulated gastric fluid. A fast release profile was observed in the initial 30 min of the gastric simulation, reaching then a first plateau until the end (120 min) probably due to the solubilization of the DNJ located on the surface of SDMs. These results highlighted that the electrostatic interaction between DNJ and ALG was stronger than DNJ salt formation with the HCl present in the gastric environment. For this reason, the complete release did not occur even though DNJ is a highly hydrophilic compound. An additional release was observed during the subsequent intestinal simulation. The pH transition from 3.0 to 6.8 might have induced the rearrangement of the polymer structure followed by the progressive solubilization of the SDMs, leading to a faster release of DNJ in the first 30 min. The compound reached a new plateau (at 150 min) without exceeding 43% of the cumulative release. The incomplete release of DNJ was in contrast with the findings of Gavini and co-workers, who evaluated the in vitro release of metoclopramide from SDMs [46]. The authors achieved the complete release of the alkaline drug in 3 h (pH 7), even though a possible electrostatic interaction between the ALG and the drug was supposed [46]. This difference might be due to the higher molecular weight and steric hindrance of metoclopramide compared to the DNJ which led to a weaker interaction with ALG chains.

Thus, the results of the in vitro simulated release suggested that Ca-ALG beads and SDMs could maintain their integrity in the acid and hostile environment of the stomach. Furthermore, the controlled release of DNJ seemed to be mainly governed by the electrostatic interaction between the active compound and the carboxylic groups of ALG. Therefore, the encapsulation of DNJ into a structured carrier such as the Ca-ALG beads might not be necessary to achieve the goal of a sustained and prolonged release.

## 3. Materials and Methods

### 3.1. Chemicals and Solvents

Acetonitrile and ethanol (HPLC grade), ammonium formate (purity > 99.0%), calcium chloride, disodium hydrogen phosphate monohydrate, deuterium oxide (D_2_O), DNJ reference standard (purity ≥ 95.0%), hydrochloric acid, and 3-(trimethylsilyl)propionic-2,2,3,3-d4 acid sodium salt (TSP) were provided by Merck Life Science S.R.L. (Milan, Italy). Sodium alginate (Na-ALG, high G/M ratio) was obtained from Honeywell Fluka (Charlotte, NC, USA). Water was purified by using a Milli-Q Plus185 system from Millipore (Milford, MA, USA).

### 3.2. Plant Material and Extraction of Mulberry Leaves

*Morus alba* (L.) leaves collected from the cultivar Nervosa were used to obtain a DNJ-rich extract. Mulberry leaves were provided by the Research Centre for Agriculture and Environment (CREA), a laboratory of sericulture located in the north-east of Italy (45°24′57″96 N lat., 11°52′58″08 E long., Padova, Italy). The harvest was carried out in the middle of 2020 summer, when the DNJ content was the highest, as was demonstrated by a previous study conducted by Marchetti and co-workers [18]. Leaves were dried in an oven at 50 °C until reaching constant weight and then ground in an automatic mill. The extraction was performed by dynamic maceration of 10 g of leaves with 500 mL 50% (*v*/*v*) ethanol at room temperature. The procedure required three consecutive extraction steps of 2 h each, the first and the second with 200 mL and the last with 100 mL. The first extract was centrifuged for 5 min at 7200× *g*, paper-filtered, and a further two extractions were performed on the residue. *Morus* leaf extracts were combined in a volumetric flask and brought to 500 mL with 50% (*v*/*v*) ethanol. The described DNJ extraction procedure had been previously optimized and validated to achieve the best yield [18]. To obtain a stable dried EXT to be incorporated in beads, ethanol was evaporated under vacuum at 50 °C, and the remaining aqueous suspension was freeze-dried (Heraeus Lyovac GT2, Leybold GmbH, Cologne, Germany).

### 3.3. Determination of DNJ by HPLC-ESI-TQ-MS Analysis

The DNJ content in both the liquid and the freeze-dried EXT was determined by an Agilent 6400 Series Triple Quadrupole. The liquid EXT was diluted with acetonitrile. The freeze-dried EXT was dissolved in 50% (*v*/*v*) ethanol at a concentration of 1 mg/mL, filtered by a 0.22 μm cellulose acetate filter, and properly diluted with acetonitrile before the injection.

The LC system was equipped with a binary pump, an autosampler, an on-line degasser, and a thermostated column compartment (Agilent Technologies, Waldbronn, Germany). MassHunter (Agilent Technologies, Waldbronn, Germany) was used for data acquisition and processing. The detection was performed using the ESI source operating in positive mode. The capillary voltage was set at +3500 V. The drying gas temperature was 300 °C; nitrogen was used as a drying (flow rate 9 L/min) and nebulizing gas (pressure 28 psi). Hydrophilic interaction liquid chromatography was performed on a HILIC Cortecs UPLC column (1.6 μm, 2.1 × 100 mm). 1-Deoxynojirimycin was eluted with a binary gradient consisting of 20 mM ammonium formate in water (A) and acetonitrile (B). The gradient profile was as follows: 0–10 min, 18% A; 11–16 min, 50% A; 16–27 min, 18% A. The flow rate was adjusted to 0.3 mL/min, and the column temperature was maintained at 25 °C. HPLC analyses were performed in triplicate. The quantification of DNJ in samples was obtained by an external standard method. The calibration curve was built by analyzing six concentrations of the reference compound in the range of 0.005–0.5 μg/mL. This method had been previously validated for linearity, precision, and accuracy [18].

### 3.4. Preparation of Ca-ALG Beads

Two different batches of beads (Ca-ALG-2% and Ca-ALG-3%) were obtained starting from 2 and 3% (*w*/*v*) Na-ALG aqueous solution, respectively, and degassed through an ultrasonic bath (Bandelin electronic GmbH, Berlin, Germany). The Ca-ALG beads were prepared by ionic gelation, according to the method proposed by Iannuccelli et al. [47] with some modifications. The loaded beads (EXT-Ca-ALG-2% and -3%) were obtained by dissolving 100 mg of dry EXT into 5 mL of Na-ALG solution. To reduce the possible leakage of DNJ during the formulation process, an appropriate amount of dry EXT was dissolved into 25 mL of 2% CaCl_2_ (*w*/*v*) solution to achieve the same concentration of DNJ as for ALG. Briefly, Na-ALG solution was dropped by using a silicon tube (inner diameter 2 mm) from a height of 6 cm into CaCl_2_ solution, under gentle magnetic stirring. The newly formed beads were left for 30 min in contact with the medium under stirring, then were recovered, washed with Milli-Q water, and dried at room temperature for at least 24 h. The occurrence and complete gelation in the inner bead core were checked under visual observation by slicing some of them. Unloaded (blank) beads were prepared in the same manner, without the introduction of the EXT. All the formulations were prepared in triplicate. To prevent the absorption of moisture, the particles were stored in a desiccator before the analyses. The yield efficiency of the formulation process was calculated as follows:

$$Yield\;(\%)=\frac{Weight\;of\;the\;obtained\;beads}{\left(Weight\;of\;NaALG+Weight\;of\;EXT\right)}$$

### 3.5. Preparation of ALG Microparticles by Spray-Drying (SDMs)

One hundred milliliters of 50% ethanolic EXT of mulberry leaves was mixed under gentle magnetic stirring with one hundred milliliters of 2% (*w*/*v*) Na-ALG aqueous solution to obtain a final solution at a concentration of 1% of Na-ALG. The solution was degassed through an ultrasonic bath and spray-dried (Büchi 190 Mini-Spray Dryer, Büchi Labortechnik, Flawil, Switzerland) under the following operating conditions: inlet temperature, 140 °C; outlet temperature, 65–70 °C; pump flow, 3 mL min^−1^; spray flow, 600 Nlh^−1^; aspirator setting, 15; nozzle cap diameter, 0.5 mm. Unloaded (blank) SDMs were obtained under the same operating conditions starting from a 1% Na-ALG solution in water. All the formulations were prepared in triplicate. To prevent the absorption of moisture, the particles were stored in a desiccator before the analyses. The yield efficiency of the formulation process was calculated as follows:

$$Yield\;(\%)=\frac{Weight\;of\;the\;obtained\;SDMs}{\left(Weight\;of\;NaALG+Weight\;of\;EXT\right)}$$

The weight of the EXT was calculated by considering the mean weight of the dry EXT obtained from the freeze-drying process.

### 3.6. Particle Size Analysis of Ca-ALG Beads and SDMs

The size of blank and loaded beads was determined by analyzing the images captured by a digital camera. Bead diameters were measured by ImageJ free license software (Version 1.5, National Institutes of Health, New York, NY, USA). ImageJ software was calibrated to transform the measured pixels in length units (mm) by measuring a caliper section. The relative frequency distribution of each sample was calculated and the data fit with a Gaussian equation in GraphPad Prism 8.4.3 (GraphPad Software, San Diego, CA, USA).

The particle size of SDMs was determined via a laser diffrasctometer (Mastersizer hydro 2000 MU, Malvern Panalytical, Malvern, UK) by suspending about 20 mg of SDMs in 25 mL of isopropanol under magnetic stirring.

The homogeneity of particles was evaluated by calculating the span value as follows:

$$Span=\frac{\left(d^{90}-d^{10}\right)}{d^{50}}$$

where *d*^10^, *d*^50^, and *d*^90^ represent the fine, the median, and the coarse particle fractions, respectively.

### 3.7. Morphological Analyses

The morphological analyses were performed by environmental scanning electron microscopy (ESEM, Quanta 200, Fei, Hillsboro, OR, USA). Before the analysis, Ca-ALG beads and SDMs were placed in a desiccator overnight to remove any residual moisture. The loaded and blank Ca-ALG beads and SDMs were fixed on aluminum stubs using a double-sided carbon tape and then vacuum-coated with gold–palladium in an argon atmosphere for 1 min (Sputter Coater Emitech K550, Emitech Ltd., Ashford, Kent, UK).

### 3.8. DNJ Loading and Encapsulation Efficiency

Ten micrograms of each preparation was placed in one milliliter of 0.2 M HCl solution to determine the amount of encapsulated DNJ in Ca-ALG beads and SDMs. The strong acid solution was selected to break the interaction between the DNJ and the polymer. After 24 h, the suspension was mixed by vortex, diluted with ethanol, and analyzed by HPLC-ESI-TQ-MS, as described in Section 2.3. The actual drug loading (*DL*) and the encapsulation efficiency (*EE*%) were calculated with the following equations:

$$DL=\frac{Loaded\;DNJ\;\left(\mu g\right)}{Particle\;weight\;\left(mg\right)}\;$$

$$EE\;\left(\%\right)=\frac{Actual\;DL}{Theoretical\;DL}\times 100$$

### 3.9. Fourier-Transform Infrared (FTIR) Spectroscopy

ATR-FTIR spectra of Ca-ALG beads and SDMs (and relative blanks) were obtained using a Spectrum Two equipped with Universal ATR sampling accessory (Perkin Elmer, Milano, Italy). Beads were crushed in a mortar to obtain a fine powder. The acquisition spectral range was 4000–450 cm^−1^ with 16 scans and a resolution of 4 cm^−1^.

### 3.10. Nuclear Magnetic Resonance (NMR) Relaxometry

NMR relaxation measurement has been largely used for the study of molecule interactions and local motions. Two relaxation time constants (T_1_ and T_1_ρ) of DNJ protons were considered for this purpose. After a pulse, the spin–lattice relaxation time (T_1_) is the time constant for the regrowth of longitudinal magnetization, namely along the direction of the main magnetic field (*z*-axis). As a result, T_1_ determines the rate at which a pulse sequence can be repeated. Instead, the spin–lattice in the rotating frame relaxation time (T_1_ρ) is the time constant for the decay of magnetization across the radiofrequency field of an applied spin-locking pulse in the rotating frame. The value of T_1_ρ is measured by first applying a 90° radiofrequency pulse to an equilibrium magnetization vector. A second pulse is then applied, which effectively locks the magnetization vector into the transverse plane (xy). During the spin-locking pulse, the magnetization vector decays to its equilibrium value, with a time constant equal to T_1_ρ. T_1_ρ is known to be more sensitive than T_1_ to slow molecular fluctuations, typical of in vivo processes such as chemical exchanges with macromolecules [48,49,50]. To assess whether a sort of interaction existed, the T_1_ρ values of DNJ were measured in the absence and presence of ALG in a molar ratio of 50:1. Afterward, to gain more insight into the results, specific interaction constants between the ligand (DNJ) at different concentrations and the polymer (ALG) were calculated, according to the method proposed by [32]. In this regard, DNJ-ALG solutions were prepared by adding 1% (*w*/*v*) ALG to DNJ solutions in D_2_O at different concentrations (4, 5, 10, 15, 20, and 25 mM). The association constants (Ka) were determined following the evolution of DNJ relaxation times by increasing its concentration and keeping that of ALG constant. NMR experiments were carried out on a Bruker FT-NMR Avance III HD 600 MHz spectrometer (Bruker Biospin GmbH Rheinstetten, Karlsruhe, Germany) at 298 K non spinning. The chemical shift values were expressed in ppm relative to TSP. T_1_ρ relaxation times were measured by using the Bruker sequence “t1rho_esgp2d”. The acquisition parameters were as follows: time domain (number of data points), 32 K; dummy scans, 4; number of scans, 16; pulse width, 12.03 μs (90°); acquisition time, 2.50 s; delay time, 5 s; spectral width, 11 ppm (6602 Hz); fid resolution, 0.40 Hz; digitization mode, baseopt. The total acquisition time was 32 min and 30 s. A total of 14 values for τ from 10 ms to 6 s were employed for the calculation. ^1^H-NMR experiments for the measurement of T_1_ were carried out using the Bruker sequence “t1ir_pr”, with presaturation of residual water signal. The acquisition parameters were as follows: time domain (number of data points), 128 K; dummy scans, 0; number of scans, 4; pulse width, 11.84 μs (90°); acquisition time, 4.96 s; delay time, 5 s; spectral width, 22 ppm (13,204 Hz); fid resolution, 0.2 Hz; digitization mode, baseopt. The total acquisition time was 7 min and 42 s. Spin–lattice relaxation times were measured by inversion-recovery pulse sequence (180°–τ–90°) and fitted by exponential regression analysis of the recovery curves of longitudinal magnetization components. A total of 8 values for τ from 100 μs to 20 s were employed for the calculation.

### 3.11. Ca-ALG Bead Swelling Study

The water absorption behavior of Ca-ALG beads was studied gravimetrically [51]. The pre-weighed beads (approximately 100 mg) were placed in 10 mL of simulated gastric fluid (HCl solution, pH 3) and 0.1 M phosphate buffer saline (PBS, pH 6.8) under sink conditions at 37 °C. The beads were taken out at fixed time intervals and wiped superficially to remove weakly bound surface water, then accurately weighed, and put again in the swelling media. All the experiments were carried out in triplicate. The dynamic weight change (%) of the beads was determined according to the following expression:

$$Weight\;change\;\left(\%\right)=\frac{Ws-Wi}{Wi}\times 100$$

where *Ws* is the weight of the beads in the swollen state and *Wi* is the initial weight.

### 3.12. In Vitro Release of DNJ

The release of DNJ from the different formulations was investigated in sink conditions in simulated gastrointestinal fluids. Regarding DNJ release from Ca-ALG beads, approximately 40 mg was placed into 4 mL of HCl solution (pH 3) under magnetic stirring at 100 rpm at 37 ± 0.5 °C, and after 2 h the beads were paper-filtered and placed in 4 mL of 0.1 M PBS (pH 6.8) for a further 3 h under continuous stirring. Regarding SDMs, an aliquot of 40 mg was placed into a dialysis membrane (cut off 12,000/14,000 Da) and immediately soaked in 10 mL of HCl solution (pH 3.0) under magnetic stirring (100 rpm) at 37 ± 0.5 °C. After 2 h, the dialysis membrane was moved into 10 mL of PBS (pH 6.8) for a further 3 h, under stirring at 37 ± 0.5 °C. For both the release studies, sample solutions were withdrawn at fixed time intervals and the initial volume was restored with fresh medium. The solutions were properly diluted with the mobile phase and analyzed by HPLC-ESI-TQ-MS. The experiments were performed in triplicate on different batches.

### 3.13. Statistical Analysis

Significant differences within groups were determined at *p* < 0.05 using Student’s *t*-test and one-way analysis of variance (ANOVA) followed by Tukey’s post hoc test. All the analyses were accomplished using GraphPad Prism 8.4.3 (GraphPad Software, San Diego, CA, USA).

## 4. Conclusions

In the current work, two different strategies for the incorporation of DNJ were developed and fully characterized to prolong the release and improve its therapeutic efficacy. Both carriers were demonstrated to successfully delay and control the release of DNJ in the gastrointestinal environment, but the highest encapsulation efficiency was achieved for SDMs. In vitro simulation pointed out that the release was governed by the strong electrostatic interaction between the iminosugar and the carboxylic groups of ALG, which was also demonstrated by NMR. The DNJ delivery strategy based on the cross-linking of Na-ALG with CaCl_2_ to generate Ca-ALG beads was shown to be the less convenient option to reach the goal. Indeed, Ca-ALG beads required a higher amount of EXT in the gelling solution to achieve the same loading compared to SDMs. In conclusion, the SDM formulation was the easiest, most promising, and scalable strategy to deliver the mulberry DNJ, although the product yield might be improved. Based on these findings, the active compound could be released slowly and gradually from the stomach to the intestine, allowing it to persist in situ and act over an extended period. This evidence suggests that a large dose of the loaded compound would pass into the bloodstream, ensuring the highest bioavailability and the optimal therapeutic result. This work is of utmost importance to overcome issues related to the poor bioavailability of DNJ. The demonstration of the electrostatic interaction between ALG and DNJ would lay the foundation for the production of more efficacious food supplements for the management of hyperglycemic conditions.

## Figures and Tables

**Table 1:** Particle size, size homogeneity, yield, entrapment efficiency (EE%), and drug loading (DL) of Ca-ALG beads and SDMs.

Formulation	Size		Distribution Width (SPAN Value)	Yield (%)	EE (%)	DL (mg/mg)
Blank Ca-ALG-2% beads	1.30 ± 0.19 mm		0.40 ± 0.02	72 ± 2	-	-
EXT-Ca-ALG-2% beads	1.10 ± 0.16 mm		0.35 ± 0.03	76.3 ± 1.8	54.0 ± 0.4	0.52 ± 0.02
Blank Ca-ALG-3% beads	1.55 ± 0.15 mm		0.27 ± 0.01	88 ± 3	-	-
EXT-Ca-ALG-3% beads	1.5 ± 0.2 mm		0.34 ± 0.07	91.6 ± 1.1	55.2 ± 0.3	0.43 ± 0.04
Blank SDMs	11 ± 6 μm		3.73 ± 0.15	65 ± 3	-	-
EXT-SDMs	80%	11 ± 5 μm	7.8 ± 1.6	66.70 ± 2.51	99 ± 3	0.63 ± 0.02
	20%	53 ± 11 μm				

Data are expressed as mean ± SD of three independent experiments.

**Table 2:** ^1^H-NMR assignments, chemical shifts, and signal multiplicity for DNJ in deuterium oxide.

DNJ Assignment	δ (ppm)	Multiplicity	J (Hz)
H1	2.47	dd	10.80, 12.50
H1	3.13	dd	5.05, 12.36
H2	3.50	m	₋
H3	3.33	t	9.14
H4	3.24	t	9.50
H5	2.56	m	₋
H6	3.64	dd	6.23, 11.64
H6	3.84	dd	2.90, 11.60

**Table 3:** Spin–lattice relaxation time in the rotating frame (T_1_ρ) of DNJ protons in the absence and presence of alginate.

DNJ	T_1_ρ DNJ (s)	T_1_ρ DNJ + ALG (s)	Δ(s)	Δ%
H1	0.535	0.440	−0.095	−17.76
H1	0.663	0.530	−0.133	−20.06
H2	1.760	1.390	−0.370	−21.02
H3	1.810	1.430	−0.380	−20.99
H4	1.801	1.401	−0.400	−22.21
H5	0.963	0.640	−0.323	−33.54
H6	0.679	0.548	−0.131	−19.29
H6	0.680	0.517	−0.163	−23.97

**Table 4:** Spin–lattice relaxation times (T_1_) of DNJ protons in the absence and presence of ALG.

DNJ (mM)	T_1_ H1 2.47 ppm (s)		T_1_ H1 3.13 ppm (s)		T_1_ H2 3.50 ppm (s)		T_1_ H3 3.33 ppm (s)		T_1_ H4 3.24 ppm (s)		T_1_ H5 2.56 ppm (s)		T_1_ H6 3.64 ppm (s)		T_1_ H6 3.84 ppm (s)	
	DNJ	DNJ + ALG	DNJ	DNJ + ALG	DNJ	DNJ + ALG	DNJ	DNJ + ALG	DNJ	DNJ + ALG	DNJ	DNJ + ALG	DNJ	DNJ + ALG	DNJ	DNJ + ALG
4	0.671	0.545	0.781	0.597	2.422	1.438	2.505	1.934	0.818	0.523	1.468	1.048	0.827	0.618	2.542	1.742
5	0.670	0.538	0.784	0.560	2.382	1.346	2.501	1.837	0.817	0.523	1.469	0.963	0.820	0.592	2.533	1.569
10	0.662	0.559	0.771	0.604	2.337	1.532	2.440	2.036	0.806	0.579	1.432	1.061	0.808	0.630	2.463	1.810
15	0.666	0.580	0.774	0.607	2.347	1.607	2.459	2.075	0.811	0.597	1.435	1.071	0.814	0.637	2.468	1.861
20	0.661	0.584	0.770	0.605	2.324	1.739	2.431	2.124	0.806	0.616	1.422	1.080	0.806	0.650	2.440	1.893
25	0.654	0.584	0.760	0.637	2.212	1.780	2.323	2.090	0.794	0.669	1.403	1.070	0.787	0.695	2.386	1.860

**Table 5:** Association constants (Ka) of DNJ protons obtained from the difference of spin-lattice relaxation time (T_1_) values in absence and presence of ALG.

DNJ (mM)	1/Δ*R* (s^−1^)							
	H1 2.47 ppm	H1 3.13 ppm	H2 3.50 ppm	H3 3.33 ppm	H4 3.24 ppm	H5 2.56 ppm	H6 3.64 ppm	H6 3.84 ppm
4	2.900	2.534	3.539	8.485	1.450	3.663	2.445	5.535
5	2.730	1.960	3.095	6.919	1.453	2.796	2.129	4.123
10	3.600	2.789	4.448	12.297	2.056	4.095	2.860	6.827
15	4.492	2.813	5.097	13.288	2.262	4.222	2.929	7.567
20	5.040	2.823	6.900	16.819	2.613	4.491	3.358	8.444
25	5.420	3.936	9.114	20.837	4.260	4.508	5.945	8.437
−1/Ka	−17.646	−28.406	−7.098	−8.365	−6.609	−49.466	−9.198	−23.948
Ka	57	35	141	120	151	20	109	42

## Data Availability

The data presented in this study are available on request from the corresponding author.
